# Chemical informatics uncovers a new role for moexipril as a novel inhibitor of cAMP phosphodiesterase-4 (PDE4)

**DOI:** 10.1016/j.bcp.2013.02.026

**Published:** 2013-05-01

**Authors:** Ryan T. Cameron, Ryan G. Coleman, Jon P. Day, Krishna C. Yalla, Miles D. Houslay, David R. Adams, Brian K. Shoichet, George S. Baillie

**Affiliations:** a Institute of Cardiovascular and Medical Sciences, CMVLS, Glasgow University, Glasgow G12 8QQ, UK; b Department of Pharmaceutical Chemistry, University of California, San Francisco, CA 94158, USA; c Institute of Chemical Sciences, Heriot-Watt University, Edinburgh EH14 4AS, UK; d Institute of Pharmaceutical Science, King's College London, Franklin-Wilkins Building, 150 Stamford Street, London SE1 9NH UK

**Keywords:** Phosphodiesterase inhibitor, Protein kinase A (PKA), PDE4, Catechol ether, Cyclic 3′5′ adenosine monophosphate (cAMP)

## Abstract

PDE4 is one of eleven known cyclic nucleotide phosphodiesterase families and plays a pivotal role in mediating hydrolytic degradation of the important cyclic nucleotide second messenger, cyclic 3′5′ adenosine monophosphate (cAMP). PDE4 inhibitors are known to have anti-inflammatory properties, but their use in the clinic has been hampered by mechanism-associated side effects that limit maximally tolerated doses. In an attempt to initiate the development of better-tolerated PDE4 inhibitors we have surveyed existing approved drugs for PDE4-inhibitory activity. With this objective, we utilised a high-throughput computational approach that identified moexipril, a well tolerated and safe angiotensin-converting enzyme (ACE) inhibitor, as a PDE4 inhibitor. Experimentally we showed that moexipril and two structurally related analogues acted in the micro molar range to inhibit PDE4 activity. Employing a FRET-based biosensor constructed from the nucleotide binding domain of the type 1 exchange protein activated by cAMP, EPAC1, we demonstrated that moexipril markedly potentiated the ability of forskolin to increase intracellular cAMP levels. Finally, we demonstrated that the PDE4 inhibitory effect of moexipril is functionally able to induce phosphorylation of the small heat shock protein, Hsp20, by cAMP dependent protein kinase A. Our data suggest that moexipril is a bona fide PDE4 inhibitor that may provide the starting point for development of novel PDE4 inhibitors with an improved therapeutic window.

## Graphical abstract

**Figure:**
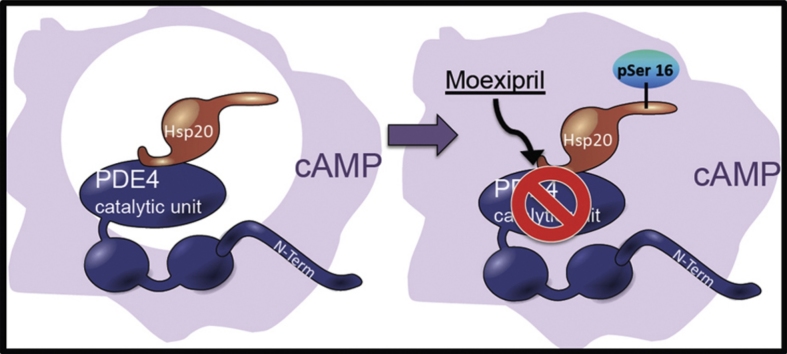

## 1 Introduction

The escalating costs and diminishing returns of drug development have fuelled a growing focus on drug repositioning in recent years [1]. As annual approvals of new molecular entities (NMEs) dwindle in the face of increasing economic and regulatory pressures [2], greater emphasis is being placed on the development of systematic approaches for identification of compounds with repositioning potential, including the application of *in silico* structure-based and chemoinformatic methodologies [3–5]. We have used such approaches to find novel inhibitors of the important cAMP hydrolyzing phosphodiesterase 4 (PDE4) enzyme family, which has been implicated in the pathophysiology underlying a range of diseases and conditions that include schizophrenia, stroke and asthma [6].

PDE4 is one of eleven known phosphodiesterase families and plays a pivotal role in mediating hydrolytic degradation of the important cyclic nucleotide second messenger, cyclic AMP (cAMP) [7]. The PDE4 family acts to regulate downstream signalling events induced by cAMP, and does so *via* the action of approximately 25 different isoforms that arise as multiple splice variants encoded by four distinct genes (*PDE4A*, *B*, *C* and *D*) [8]. The fact that all PDE4 enzymes have been highly conserved over evolution suggests that they have non-redundant functional roles in regulating cAMP homeostasis linked to the compartmentalisation of cAMP signalling [9]. As all PDE4 isoforms have similar *K*_m_ and *V*_max_ parameters for cAMP hydrolysis, their functional roles are determined largely by their cellular location and post-translational modification. Discrete intracellular targeting of individual PDE4 isoforms is most often directed by a “postcode” sequence within their unique N-terminal domains [10], which are responsible for promoting many of the protein–protein and (in one case) protein–lipid interactions that act to anchor PDE4s to signalling nodes in sub-cellular compartments [6].

**Fig. 1:**
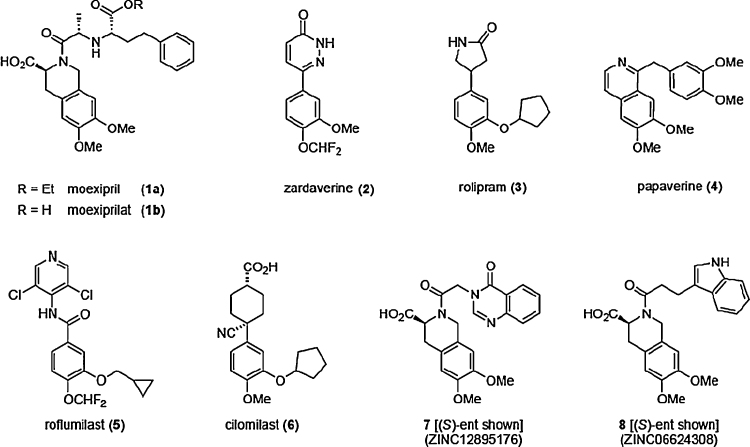
Established PDE4 inhibitors (**2**–**6**) and newly identified PDE4-inhibitory 3-carboxy-6,7-dimethoxytetrahydroisoquinoline compounds: moexipril (**1a**), **7** and **8**.

It is well established that inhibitors targeted to the catalytic pocket of PDE4s show promise for the treatment of chronic obstructive pulmonary disease (COPD) and asthma, rheumatoid arthritis, inflammatory bowel disease and psoriasis [11,12]. PDE4 inhibitors have also been shown to be effective in reversing age associated memory deficits, promoting memory function and treating depression [13]. Thus, in principle, PDE4 inhibitors have considerable therapeutic potential. In practice, however, their clinical utility has been compromised by mechanism-associated side effects that limit maximally tolerated doses [14]. Headache, nausea, emesis and diarrhoea are the most commonly reported side effects and these stem from the inhibition of PDE4 activity in non-target tissues. In particular, PDE4D expression is high in a region of the brain, the area postrema, where inhibitor action may trigger nausea [14]. Despite the challenges to therapeutic deployment of PDE4 inhibitors, one such compound (roflumilast, Fig. 1) has recently been approved by the European Commission and US Food and Drug Administration (FDA) for the treatment of severe COPD [15], albeit that concern remains over side-effects such as diarrhoea, pancreatitis and weight loss associated with its administration [16].

One strategy to develop a novel, safer class of PDE4 inhibitor would be to survey existing approved drugs for PDE4-inhibitory activity. With this objective we have used a high-throughput computational approach to identify moexipril (**1a**, Fig. 1), a well tolerated and safe angiotensin-converting enzyme (ACE) inhibitor [17], as a PDE4 inhibitor. Moexipril may thus, in principle, constitute a new starting point for development of pharmacologically useful PDE4 inhibitors.

## 2 Materials and methods

### 2.1 Chemical informatics

The 2010 MDL Drug Data Report was used as a source of on market drugs [18] and each drug was compared to the sets of ligands for each PDE4 subtype according to ChEMBL [19] with the Similarity Ensemble Approach [5,20]. The ACE inhibitor moexipril [21,22] was identified as a potential PDE4A, B, C and D inhibitor by SEA, with an *E*-value of 1.71^−11^ and a max Tanimoto coefficient in ECFP4 fingerprints of 0.35. Moexipril was tested for colloidal aggregation [23] by dynamic light scattering where no particles were observed, additionally it did not inhibit beta-lactamase at 10 or 100 μM. Searching for analogues of moexipril was done with ZINC [24]. Docking to PDB Code 1MKD [25] was performed with DOCK3.6 [26], the best scoring pose that overlapped the known ligand was chosen.

### 2.2 Chemicals

Moexipril, rolipram, KT5720, forskolin, IBMX were bought from Sigma–Aldrich (UK). Compounds **7** and **8** were purchased from Princeton BioMolecular Research (USA). All cell culture media, sera and solutions were purchased from Gibco (Invitrogen Life Technologies, UK)

### 2.3 Cell culture

HEK293 cells were maintained in DMEM containing 10% (v/v) FBS, 2 mM l-glutamine and 1% penicillin/streptomycin. SH-SY5Y cells were maintained in DMEM:F12 (1:1) containing 10% (v/v) FBS, 2 mM l-glutamine and 1% penicillin/streptomycin. For FRET analysis SH-SY5Y cells stably expressing Epac1-camps under G418 selection (500 μg/ml) were seeded onto 22 mm round glass coverslips and maintained in a 6 well plate 24 h prior to use.

### 2.4 Transient expression of PDE4 isoforms in HEK293 cells

Expression plasmids encoding human PDE4 were as previously described by us [27,28]. The plasmids were purified from *Escherichia coli* using the Maxi-prep system (Qiagen, UK). For transient transfections, HEK293 cells were seeded at a 1:3 ratio into culture flasks 24 h before transfection so that cells were ~60% confluent by the time of transfection. Transfections were carried out using PolyFect^®^ transfection reagent (Qiagen, UK) in accordance with manufacturer's instructions.

### 2.5 Generation of HEK293 cell lysates for PDE assay

Cells (~90–100% confluent) were transfected for 48 h with cDNA encoding PDE4B2, PDE4A5, PDE4D5, PDE8A and PDE5, washed with PBS and harvested by using a cell scraper in KHEM buffer (50 mM KCl, 50 mM HEPES; pH 7.2, 10 mM EGTA, 1.92 mM MgCl_2_, 1 mM dithiothreitol (DTT)) supplemented with protease inhibitor Mini-Complete (Roche, UK). Samples were then frozen on solid CO_2_, thawed and then manually homogenised, followed by passage through a 26-gauge needle several times to ensure complete cell lysis. Cells were centrifuged at 13,000 rpm for 10 min to remove any unbroken cells, and the resulting supernatant was frozen in solid CO_2_ and stored at −80 °C until required. For experimentation, the protein concentration of whole-cell lysate from transfected and mock-transfected (vector only) cells was equalised (typically to 1 μg/μl). Protein concentration was determined through Bradford Assay using bovine serum albumin as standard.

### 2.6 PDE assays

PDE activity was determined using a two-step radioassay procedure as described previously [29]. Activities for each PDE subtype were related to a non-drug treated sample (100% control) over an increasing dose of the indicated compounds. IC_50_ values were calculated using. In all cases, the transfected PDE accounted for over 97% of the total PDE activity when compared with the untransfected control lysates.

### 2.7 FRET imaging

FRET imaging experiments were performed on SH-SY5Y-Epac1-camps stables. Cells were maintained at room temperature in DPBS (Invitrogen, UK), with added CaCl_2_ and MgCl_2_, and imaged on an inverted microscope (Olympus IX71) with a PlanApoN, 60X, NA 1.42 oil, 0.17/FN 26.5, objective (Japan). The microscope was equipped with a CCD camera (cool SNAP HQ monochrome, Photometrics), and a beam-splitter optical device (Dual-channel simultaneous-imaging system, DV^2^ mag biosystem (ET-04-EM)). Imaging acquisition and analysis software used was Meta imaging series 7.1, Metafluor, and processed using ImageJ (http://rsb.info.nih.gov/ij/). FRET changes were measured as changes in the background-subtracted 480/545-nm fluorescence emission intensity on excitation at 430 nm and expressed as either *R*/*R*0, where *R* is the ratio at time *t* and *R*0 is the ratio at time = 0 s, or Δ*R*/*R*0, where Δ*R* = *R* − *R*0. Values are expressed as the mean ± SEM.

### 2.8 Hsp20 phosphorylation assay

SH-SY5Y cells were seeded at a density of 1 × 10^6^ cell per well onto 6 well plates (Corning, UK) for at least 16 h prior to treatment with rolipram (10 μM), moexipril (50 μM), compound **7** (50 μM) and compound **8** (50 μM). Compounds were diluted in media and added to cells for 0.5, 1 and 2 h prior to harvesting using 3T3 lysis buffer (1% Triton X-100, 50 mM Hepes, pH 7.2, 10 mM EDTA and 100 mM NaH_2_PO_4_) supplemented with protease inhibitor Mini-Complete (Roche) and phosphatase inhibitor PHOS-stop (Roche, UK). Hsp20 expression was analysed using standard SDS-PAGE and Western Blotting techniques using the phospho-Hsp20 antibody (ab58522 Abcam, UK) and alpha-tubulin-HRP antibody (ab40742 Abcam, UK) as the loading control. Western blotting for PDE4s was undertaken using antibodies previously described by us [30].

## 3 Results

### 3.1 Chemical informatics and docking studies identify moexipril as a candidate PDE4 inhibitor

The similarity ensemble approach (SEA) is one of a number of *in silico* methods now used to identify off-target activity in drugs. The technique measures the topological similarity between bait molecules, here for instance moexepril, and a set of ligands annotated to any given target in a library of target-ligand sets. The observed similarities between the bait molecule(s) and the ligand-sets are compared to what would be expected at random, and the expectation value of seeing the level of similarity observed is calculated [4,5]. Because SEA compares molecules to annotated ligands as sets, collective similarity can be established even when the pair-wise similarity to any single ligand in the set may be modest. It has been applied successfully to predict activity of established drugs against previously unreported targets [4,31] and also used to predict biological activity in natural products [32]. Here we applied SEA to probe the MDL Drug Data Report (MDDR), a database currently comprising >180,000 biologically relevant compounds with a focus on drugs that are launched or under current development. In doing so we identified moexipril [21,22,33] as a candidate PDE4 inhibitor, using ChEMBL to examine known sets of PDE4 active compounds [19]. Though moexipril's similarity to even the closest known PDE4 inhibitor was modest – a Tanimoto coefficient of 0.35 qualifies it as close to a scaffold-hop for the ECFP4 fingerprints [Ref.: PMID 18416545] – over the entire PDE4 ligand set its expectation value (*E*-value), at 1.71^−11^, was highly significant compared to the random background.

### 3.2 Models of moexipril bound to catalytic domain of PDE4

**Fig. 2:**
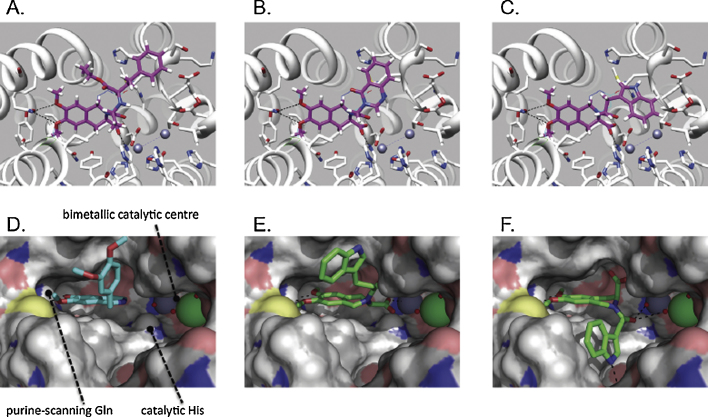
Docked models of newly identified 3-carboxy-6,7-dimethoxytetrahydroisoquinoline inhibitors [moexipril (**1a**), **7** and **8**] fitted to the PDE4 catalytic pocket and comparison with papaverine (**4**). (A)–(C) Best scoring poses for moexipril, **7** and **8** docked into the PDE4 zardaverine co-crystal structure (PDE4: 1MKD). (D) Structure of papaverine (cyan stick) bound to PDE4D core catalytic domain (PDB: 3IAK). (E) and (F) models of inhibitor **8** (green stick) fitted to the PDE4 papaverine co-crystal structure showing poses with alternative conformations for the tetrahydroisoquinoline core. (For interpretation of the references to colour in this figure legend, the reader is referred to the web version of the article.)

As the structure of the PDE4 core catalytic domain is well defined by X-ray crystallography, with numerous co-crystal structures available for a range of inhibitors from different structural classes, we additionally undertook the molecular docking of moexipril to consider its potential as a PDE4 inhibitor. Docking was carried out with DOCK3.6 [26] against the co-crystal structure (PDB: 1MKD) of the PDE4D core catalytic domain with bound zardaverine (**2**) [25]. In the best scoring pose (Fig. 2A), the 6,7-dimethoxytetrahydroisoquinoline core of moexipril overlapped closely with the catechol ether subunit of zardaverine (**2**) to engage the purine-scanning glutamine, a residue that is conserved across the entire PDE superfamily and which ordinarily anchors the substrate nucleobase during enzymatic turnover. Catechol ethers such as zardaverine [34] constitute one of the main PDE4 inhibitor chemotypes and include rolipram (**3**) [35], the archetypal PDE4-selective inhibitor, as well as the isoquinoline natural product, papaverine (**4**) [36]. The recently approved first-in-class PDE4 inhibitor, roflumilast (**5**) [37], and other compounds such cilomilast (**6**) [38] that have progressed to clinical trials also possess a catechol ether core structure. Numerous co-crystal structures are available for this class of PDE4 inhibitor [25,39,40], and in all cases the catechol ether oxygen atoms straddle the Nε centre of the purine-scanning Gln, forming convergent hydrogen bonds in the manner predicted for the docked moexipril model. The 3-carboxy group of the ligand in this pose would be orientated proximal to the bimetallic catalytic centre of the enzyme, whilst the side chain extension would be free to run across the hydrophobic rim of the catalytic pocket with little constraint.

### 3.3 Biochemical determination moexipril potency as PDE4 inhibitor

**Fig. 3:**
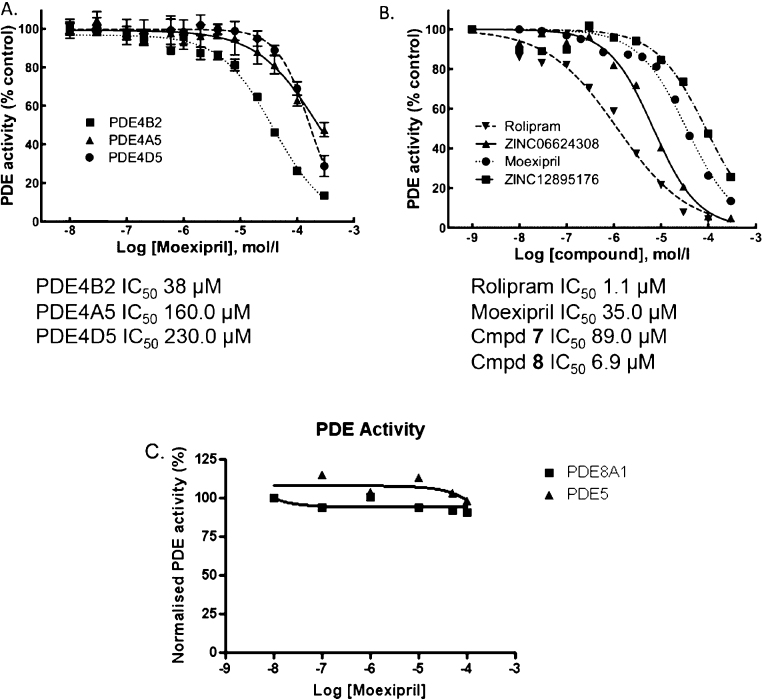
Determination of the efficacy of established and novel PDE4 inhibitors. Activities for each PDE4 subtype were related to a non-drug treated sample (100% control) over an increasing dose of the indicated compounds (*n* = 3). IC_50_ values were calculated using Graphpad Prism 4.0. (A) Dose response curves of moexipril against 3 different PDE4 isoforms. (B) Dose response curves of four different PDE4 inhibitors against PDE4B2. (C) Dose response curves of moexipril against PDE8A1 and PDE5.

To test the prediction that moexipril might exhibit PDE4-inhibitory activity, we assayed the compound for inhibition of three widely expressed PDE4 isoforms PDE4A4, PDE4B2 and PDE4D5. Moexipril inhibited cAMP hydrolysis by all three isoforms in the micromolar range (Fig. 3A), but was most potent against the PDE4B2 isoform (IC_50_ 38 μM), with PDE4A4 and PDE4D5 showing respectively 4-fold and 6-fold lower sensitivity to inhibition. Having confirmed the prediction that moexipril should inhibit PDE4, we next undertook a search for other commercially available 3-carboxy-6,7-dimethoxytetrahydroisoquinolines using ZINC [24]. Our search identified two compounds (**7** and **8**) possessing the tetrahydroisoquinoline core of moexipril but with simplified N-acyl extensions. Both compounds were available in racemic form from screening vendors and initial docking studies, undertaken with the (*S*)-configured structures, suggested that the PDE4 catalytic pocket should be able to accommodate these compounds, with the N-acyl side chains extending across its rim (Fig. 2B and C). The (*S*)-enantiomers were selected for docking in order to match the absolute configuration at the tetrahydroisoquinoline 3-position of moexipril. The inhibitory activity of (*rac*)-**7** and (*rac*)-**8** was then assessed using PDE4B2, selected as the isoform that exhibited greatest sensitivity to inhibition by moexipril, Fig. 3B. The archetypal inhibitor, rolipram (**3**), was included in this comparative evaluation as a positive control. Consistent with the modelling, both of the moexipril analogues inhibited PDE4B2. Compound **8** showed the highest affinity for PDE4, having an IC_50_ of 6.9 μM, 7-fold better than moexipril, while compound **7** had an IC_50_ 89 μM. The inhibition curves suggest a binding mode that is competitive with cAMP for the catalytic site of the enzyme, consistent with the docked models (Fig. 2). By comparison, (*rac*)-rolipram, a drug optimised for this enzyme, had an IC_50_ 1 μM against it. Moexipril showed no activity against two other PDE family members, PDE8A and PDE5, suggesting that it could act as a PDE4 specific inhibitor (Fig. 3).

### 3.4 Moexipril induces cAMP increase in cells

**Fig. 4:**
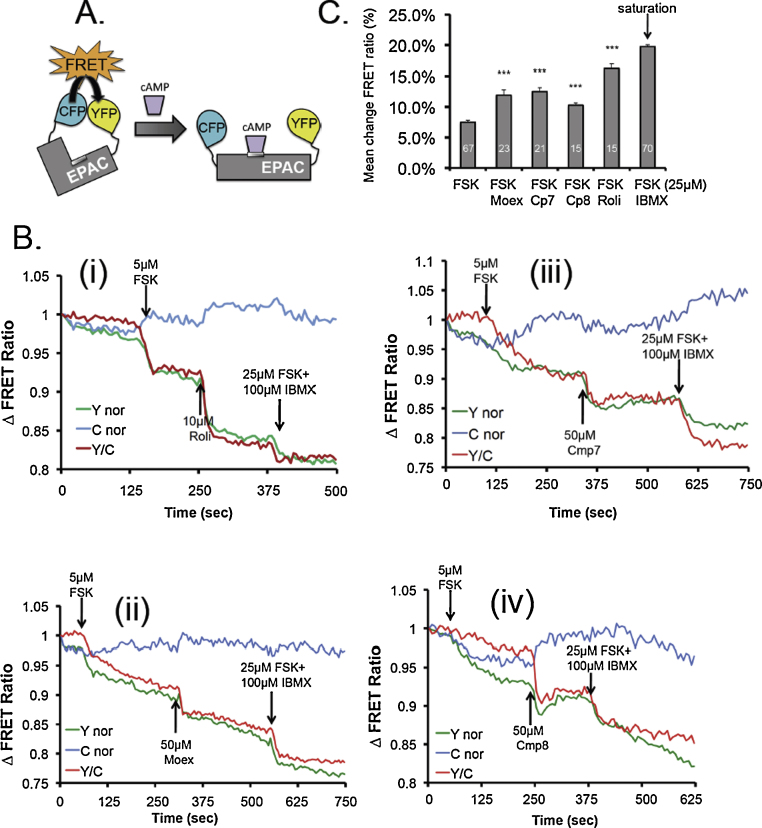
Utilisation of a cAMP reporter construct to visualise changes in cAMP concentration triggered by PDE4 inhibitors. (A) Diagram illustrating mode of action of a FRET-based biosensor constructed from the nucleotide binding domain of the type 1 exchange protein activated by cAMP, EPAC1. (B) Changes in FRET ratio triggered by a 5 μM application of forskolin (FSK), followed by treatment with PDE4 inhibitors (i) rolipram (Roli), (ii) moexipril (Moex), (iii) compound **7** (Cmp 7), and (iv) compound **8** (Cmp 8). Data is from a single cell and is representative of experiments carried out at least *n* = 15. (C) Quantification of mean change in FRET ratio for all of the treatments including in lane 6 a saturating dose of forskolin (25 μM) plus the general PDE inhibitor 3-isobutyl-1-methylxanthine (IBMX 100 μM). All other lanes forskolin (FSK) applied at 5 μM. Significance evaluated using Student's *t*-test, ***p < 0.001 when compared with FSK alone. Number of individual experiments denoted by white numbers within grey bars.

**Fig. 5:**
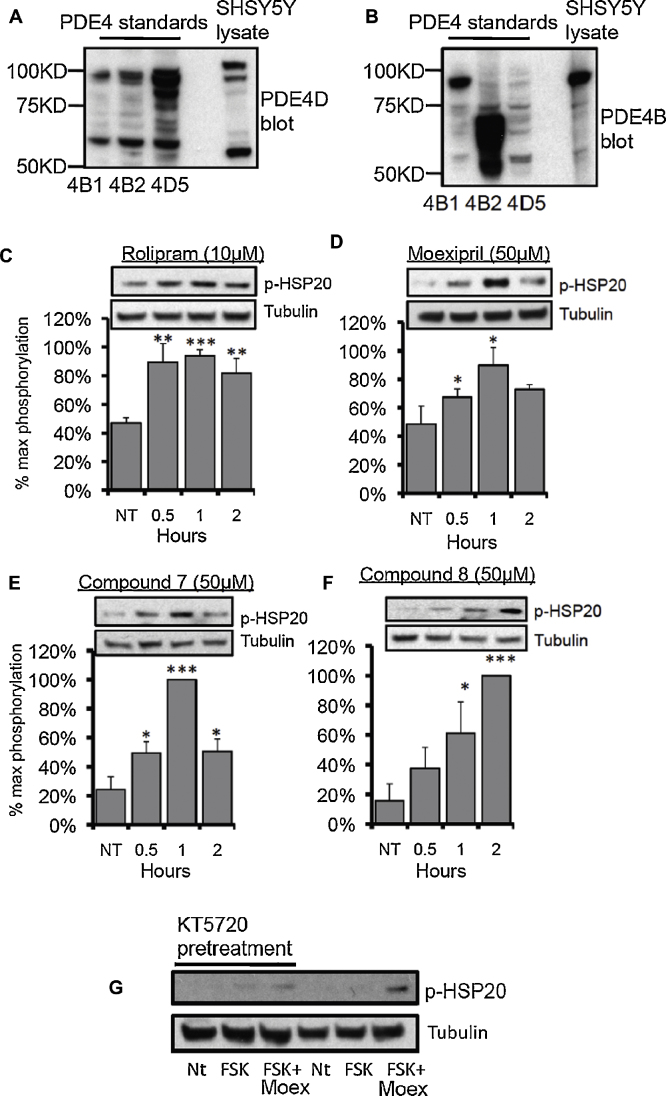
PDE4 inhibitors induce PKA phosphorylation of the small heat-shock protein Hsp20. Lysates from SH-SY5Y cells were blotted for the expression of endogenous (A) PDE4D and (B) PDE4B enzymes. SH-SY5Y cells were treated with (C) rolipram (10 μM), (D) moexipril (50 μM), (E) compound **7** (50 μM) and (F) compound **8** (50 μM) for the indicated times. Cell lysates subjected to SDS page and western blotting. Blots were probed for phospho-serine 16 on Hsp20 and a loading control (tubulin). Quantification (*n* = 3) of the relative amounts of phosphorylation on serine 16 *vs* loading control were calculated following densitometry. Results are plotted as a percentage of the maximal phosphorylation over time. Significance evaluated using Student's *t*-test, *p < 0.05, **p < 0.01, ***p < 0.001. (G) SH-SY5Y cells were treated with KT5720 (4 μM) 20 min before the addition of a sub-optimal dose of forskolin (FSK, 10 μM) or forskolin (FSK, 10 μM) with moexipril (Moex, 50 μM) for 5 min. Lysates were blotted for tubulin or phospho-serine 16 on Hsp20. Data representative of *n* = 3.

To determine whether the inhibition of PDE4 by moexipril and its analogues (**7** and **8**) could induce cellular increases in cAMP, we employed a FRET-based biosensor constructed from the nucleotide binding domain of the type 2 exchange protein activated by cAMP, EPAC1 [41] (see Fig. 4A). This probe enables quantitative, real-time detection of rapid changes in bulk cAMP following cell treatment. Experiments were done using SH-SY5Y cells stably expressing the biosensor. This cell line endogenously expresses PDE4 isoforms from the PDE4B and PDE4D subfamilies (Fig. 5A and B) [42]. All compounds markedly increased cellular cAMP levels over those induced by treatment with a sub-maximal dose of the adenylyl cyclase activator, forskolin alone (Fig. 4B–E). No FRET changes were detected when the compounds were added alone. The FRET ratio changes we observe here (Fig. 4F), are in line with those previously published for rolipram potentiation of the forskolin-stimulated cAMP response [41]. That the magnitude of cAMP response produced by moexipril and analogues evaluated here, is similar to that produced by rolipram further supports the notion that the ACE inhibitor could, in principle, also act as an *in vivo* PDE4 inhibitor.

### 3.5 Moexipril treatment triggers PKA phosphorylation of Hsp20

To evaluate whether, under the conditions of our *in vitro* studies, the elevation in global cAMP triggered by moexipril and analogues also resulted in downstream signalling events driven by the cAMP-effector protein, protein-kinase A (PKA), we studied a phosphorylation event recently attributed to the kinase. The small heat shock protein Hsp20 (HspB6) is a chaperone protein, which combats a number of pathophysiological processes in the heart, vasculature and brain [43]. The protective actions of Hsp20 require its phosphorylation by PKA on serine 16. Its association with PDE4 [44], however, keeps cAMP levels surrounding Hsp20 low, maintaining Hsp20 in its basal, unphosphorylated state. Thus association with PDE4 prevents inappropriate phosphorylation and activation of Hsp20 by fluctuations in basal cAMP levels. A similar protective ‘gating’ effect through PKA sequestration has been observed for AKAP-anchored PKA in the centrosome [45].

The PKA phosphorylation of Hsp20 was chosen here as a readout for physiological PDE4 inhibition as it has been shown previously that PDE4 inhibition alone *via* the action of rolipram, could trigger this phosphorylation event without the need for artificially raising cAMP with sub-optimal doses of forskolin to activate adenylyl cyclase [44]. We thus monitored the transient phosphorylation status of Hsp20 in SH-SY5Y cells following treatment of cells with either rolipram, or moexipril, or moexipril analogues **7** and **8** (Fig. 5C, D, E and F respectively). As previously observed with rolipram treatment [44], challenge of cells with any of three 3-carboxy-6,7-dimethoxytetrahydroisoquinoline analogues significantly elevated Hsp20 phosphorylation. The temporal nature of Hsp20 phosphorylation induction differed somewhat between compounds. However, this is likely to reflect differences in their potency in elevating cAMP levels, where rolipram induces the largest increase in cAMP (Fig. 4F) and triggers the most rapid Hsp20 phosphorylation (Fig. 5A). The transient nature of phosphorylation following treatment is likely to be attributed to compensatory mechanisms employed by the cell to combat cAMP increases, mechanisms that include activation of PDE4 enzymes by PKA [28] and dephosphorylation of Hsp20 by as yet unknown phosphatases. To prove that the observed phosphorylations were PKA dependent, a PKA specific inhibitor (KT5720) was used to attenuate the phosphorylation of HSP20 induced by moexipril and a sub-optimal dose of forskolin (Fig. 5G).

## 4 Discussion

Moexipril (**1a**) was originally developed as a long-acting, nonsulfhydryl angiotensin-I converting enzyme (ACE) inhibitor suitable for once-daily administration [33]. The drug is used to treat hypertension and is well tolerated, apparently lacking emetogenic activity [21,22]. Although moexipril itself has ACE-inhibitory activity in its own right, it serves as a prodrug for the more potent metabolite, moexiprilat (**1b**, Fig. 1), generated *in vivo* by hydrolysis of the side chain ester. PDE4-inhibitory activity has not previously been attributed to moexipril, but we identified the compound as a candidate PDE4 inhibitor by screening the MDDR drug database using the chemoinformatics SEA method. This prediction was further supported by molecular docking studies. These suggested that moexipril may feasibly bind to the PDE4 catalytic pocket with its methoxy groups engaging the purine-scanning Gln in a manner similar to the binding mode adopted by the catechol ether class of PDE4 inhibitors. Indeed moexipril is structurally related to the 6,7-dimethoxyisoquinoline natural product, papaverine (**4**), an established phosphodiesterase inhibitor of the catechol ether class for which a PDE4 co-crystal structure (PDB: 3IAK) has been determined (Fig. 2D).

To test the prediction that moexipril may inhibit PDE4 we evaluated its effect in assays using PDE4A4, PDE4B2 and PDE4D5, three ubiquitously expressed isoforms of the PDE4 family [6]. Encouragingly, our initial assessment confirmed that moexipril possesses PDE4-inhibitory activity in the enzyme assays (but not against PDE8A or PDE5). Furthermore, the inhibition of endogenous PDE4 isoforms by moexipril was evaluated using a cytosolic Epac-based FRET probe and was shown to significantly enhance intracellular cAMP increases triggered by forskolin treatment. Epac-based FRET probes require association of only one cAMP molecule to alter FRET ratios by up to 30% and they also exhibit fast activation kinetics that allow “realtime” evaluation of cAMP dynamics [46]. As the probes are not localised to any intracellular domains [41], the readout reflects changes in “global” cAMP concentrations and this is appropriate as we show that moexipril has activity against multiple PDE4 isoforms (Fig. 3) that are known to target, *via* unique N-terminal sequences, multiple and distinct cellular locations [9,10].

To demonstrate that cAMP increases initiated by the action of moexipril on PDE4s could result in downstream physiological consequences in cells, we monitored changes in the phosphorylation of a well-characterized PKA substrate, Hsp20 [43]. Hsp20 is readily phosphorylated by PKA as it exists in a complex with the A-kinase anchoring protein (AKAP), AKAP-Lbc [47]. However the activity of this Hsp20 anchored pool of PKA is tonically inhibited by sequestered PDE4 that also interacts with Hsp20 [44]. These features make Hsp20 uniquely sensitive to PKA phosphorylation following PDE4 inhibition, even under basal cAMP conditions. Both rolipram and moexipril significantly increased phospho-Hsp20 levels when compared with untreated cells, though the maximal effect was reached earlier with rolipram (Fig. 5). This is consistent with the other data we present, showing that rolipram challenge results in larger cellular increases in cAMP than does moexipril (Fig. 4F).

Moexiprilat (**1b**) was not readily available commercially and consequently we were unable to evaluate it for PDE4-inhibitory activity. Instead we searched for other commercially available 3-carboxy-6,7-dimethoxytetrahydroisoquinoline analogues in order to expand the study. Two compounds (**7** and **8**) were identified with no prior literature or patent associations and thus no previously reported biological or pharmacological activity. The compounds were sourced and tested in racemic form using PDE4B2, the latter chosen because, of the three isoforms used in our preliminary study, it had proven most sensitive to inhibition by moexipril. Indeed, both compounds showed activity, with analogue **8** exhibiting low micromolar potency (Fig. 3). In keeping with their ability to inhibit PDE4, both compounds also significantly enhanced intracellular cAMP increases triggered by forskolin challenge (Fig. 4) and induced Hsp20 phosphorylation (Fig. 5).

Docking of the (*S*)-enantiomers of both **7** and **8** confirmed that the 3-carboxy-6,7-dimethoxytetrahydroisoquinoline could fit the PDE4 catalytic pocket, whilst allowing the N-acyl side chain to roam over the hydrophobic rim of the pocket. As compounds **7** and **8** were sourced in racemic form, we cannot say to what extent the activity resides with the (*S*)-configured 3-carboxytetrahydroisoquinoline ring. Our preliminary docking studies have suggested that both enantiomers of **7** and **8** might potentially be accommodated in the PDE4 catalytic pocket and further studies would, therefore, be required to evaluate the eudismic ratio for these compounds. This is potentially an important point because the absolute configuration at the C-3 stereocentre of the tetrahydroisoquinoline core could significantly affect any ACE-inhibitory activity displayed by these simplified by moexipril analogues. Thus, although there is currently no ACE moexiprilat co-crystal structure available, inspection of co-crystal structures for closely related ‘pril’ family ACE inhibitors, such as enalaprilat (PDB: 1UZE) [48], suggests that ACE inhibition should show strong dependence on the absolute (*S*)-configuration for the moexipril(at) tetrahydroisoquinoline core. In particular, the carboxyl group of enalaprilat is directed into a pocket lined by Gln, Tyr and Lys residues that form tight hydrogen bonded and salt bridge interactions. Access to this pocket will be dependent on the absolute configuration of the stereocentre in the moexipril(at) tetrahydroisoquinoline subunit. The side chain carboxylate of moexiprilat is also expected to make a strong contribution to the compound's ACE-inhibitory activity, as (by analogy to enalaprilat) it should serve as a ligand to the zinc(II) catalytic centre of the enzyme. Thus, simplification of the N-acyl extension in compounds **7** and **8** is expected to substantially reduce any ACE-inhibitory behaviour. In short, the nature of the N-acyl side chain as well as the absolute configuration of the 3-carboxy-6,7-dimethoxytetrahydroisoquinoline core is likely to have a profound influence on ACE inhibition, and these features might be exploited to develop related compounds as PDE4 inhibitors without ACE-inhibitory activity. We have not tested **7** and **8** for ACE inhibition in the present study however.

The nature of the N-acyl side chain clearly also exerts a significant influence over the PDE4-inhibitory performance of the compounds that we have identified here. At present we cannot precisely rationalise this because the side chain extends from the opening of the catalytic pocket (Fig. 2E and F) and there is some flexibility in the potential contact that it might make with the protein. The rim of the PDE4 catalytic pocket presents an extensive hydrophobic surface, and many inhibitors with extensions projecting from a core bound within the pocket fold across this sticky surface, as illustrated in Fig. 2D for papaverine (where the pendent dimethoxybenzyl side chain fulfils this role).

In addition to the ambiguity regarding the position adopted by the side chain in the PDE4-bound state, there may be more than one conformation possible for the N-acyl tetrahydroisoquinoline core. The best scored binding poses generated from the modelling software (DOCK) orientated the 3-carboxyl group proximal to the enzyme's catalytic metal ions (Fig. 2A–C). With this organisation the ionised carboxylate might directly act as a ligand on the more deeply sited (zinc) ion or potentially hydrogen bond to water ligands on the metal centres. The adoption of this bound pose, illustrated for compound **8** in Fig. 2E, introduces a degree of strain into the tetrahydroisoquinoline subunit. An alternative conformer, with less ring strain, would possess a pseudoaxial carboxyl group, as shown in Fig. 2F. In this case the N-acyl group is predicted to hydrogen bond to water ligands on the metal centres and also to the proximal His residue (labelled in Fig. 2D) that plays a role in PDE4 catalysis by protonating the nucleotide 3′-O during substrate turnover. We cannot at present definitively indicate which of these two possibilities will be favoured for the bound compounds. The binding pose presented in Fig. 2F positions the carboxyl group into a hydrophobic subpocket in the roof of the substrate binding site, but it offers a significantly more relaxed conformation to the tetrahydroisoquinoline. In principle, with this conformation, replacement of the polar carboxyl group by a small hydrophobic substituent might enhance the affinity and PDE4-inhibitory potency of the compound, and we have previously used precisely this design principle in the development of another PDE4 inhibitor series [49].

Given the PDE4-inhibitory activity exhibited by moexipril, it is not entirely clear why the compound apparently lacks the typical side effects associated with PDE4 inhibitors. This could be due to its ADME properties, since neither moexipril nor moexiprilat is brain-penetrant. However, the dosing window may also play a role in the reported tolerance of moexipril. Thus, in one PK assessment *C*_max_ for moexipril was determined at 25 μg/L (~50 nM) from an oral dose of 15 mg, clinical trials having focused on once-daily dosing regimens in the 7.5–30 mg range. The negative charge character of the ionised moexipril and moexiprilat structures may be a contributory factor underlying their poor uptake by the brain, as with the carboxyl-bearing second generation PDE4 inhibitor, cilomilast (**6**), for which brain penetration is also limited [50]. Thus, retention of the 3-carboxyl group may be a consideration if a non-emetogenic PDE4 inhibitor series is to be developed from moexipril.

A key underlying driver behind the work described here was to identify previously approved drugs that lack any emetogenic liability as PDE4 inhibitors. Such compounds might either have direct potential for repositioning as PDE4 inhibitors or provide the starting point for development of novel PDE4 inhibitors with an improved therapeutic window. Given that the reported potency for inhibition of ACE by moexipril [IC_50_ 40 nM *vs* porcine serum ACE [21,22]] is some three orders of magnitude greater than for the inhibition of PDE4 that we disclose here, direct repositioning of moexipril for indications that might respond to treatment by PDE4 inhibitors is likely to be problematic. Not least because the profoundly higher concentrations needed to achieve PDE4 inhibition, compared to those required for ACE inhibition, may serve also to uncover an emetic response in moexipril. Nevertheless, moexipril might constitute a starting point for novel PDE4 inhibitor development, provided that derivatives can be made that lack an emetogenic profile.
